# Supplementary material for: Data-Based Variables Used as Indicators of Dairy Cow Welfare at Farm Level: A Review
Source: Animals (Basel). 2021 Dec 4;11(12):3458. doi: 10.3390/ani11123458 (PMC8698003; doi:10.3390/ani11123458)
Supplement: Supplementary file 1 [file animals-11-03458-s001.zip › animals-1387948-supplementary.pdf]

Table S1. Associations between data−based variables (DBVs) based on cow age and animal−based measurements (ABMs) extracted from two scientific publications.

DBV	ABM	Type¹ and direction²	Reference
Average age	Frequency of head butts	{~}	[1]
	Qualitive Behavior Assessment	{~}	[1]
	Cows with vulvar discharge	{−}	[1]
Average age of cattle >2 years	Severely lame cows	( )	[2]
Cows ≥2nd lactation (%)	Dirty young stock	{+}	[3]
Cows aged >5 years (%)	Cows with hairless patches	{~}	[1]
	Cows lying outside lying area	{−}	[1]
	Cows with nasal discharge	{−}	[1]
	Cows with vulvar discharge	{+}	[1]

[1] Type of associations indicated by ( ) for univariable associations and { } for multivariable associations.
[2] Direction of associations indicated by + for positive associations, − for negative associations, and ~ for nonlinear associations, empty brackets indicate that no direction was given.

Table S2. Associations between data−based variables (DBVs) addressing herd disease status or herd biosecurity status and animal−based measurements (ABMs) extracted from two scientific publications.

DBV	ABM	Type¹ and direction²	Reference
Certified disease−free «BVD»	Frequency of displacements	{+}	[1]
	Cows with dirty hindquarter	( )	[2]
	Collisions with stall components	{−}	[1]
	Time needed to lie down	{+}	[1]
	Cows with diarrhoea	{+}	[1]
	Cows with vulvar discharge	{−}	[1]
Certified disease−free «IBR″	Lean or very lean cows	( )	[2]
	Cows with diarrhoea	{+}	[1]
Certified disease−free «Salmonella″	Severely lame cows	{−}	[1]
	Dystocia %	{−}	[1]
Herd biosecurity status (open/closed)	Cows with dirty hindquarter	( )	[2]
	Severely lame cows	( )	[2]

Abbreviations: BVD = Bovine Virus Diarrhea, IBR = Infectious Bovine Rhinotracheitis
[1] Type of associations indicated by ( ) for univariable associations and { } for multivariable associations.
[2] Direction of associations indicated by + for positive associations, − for negative associations, empty brackets indicate that no direction was given.

Table S3. Associations between data−based variables (DBVs) addressing farm net results and animal−based measurements (ABMs) extracted from two scientific publications.

DBV	ABM	Type¹ and direction²	Reference
Net result (€)	Frequency of displacements	( )	[2]
	Cows with dirty hindquarter	( )	[2]
Change in net result (%)	Cows with dirty legs	{−}	[1]
	Cows with diarrhoea	{−}	[1]

[1] Type of associations indicated by ( ) for univariable associations and { } for multivariable associations.
[2] Direction of associations indicated − for negative associations, empty brackets indicate that no direction was given.

Table S4. Associations between data−based variables (DBVs) addressing culling reasons and animal−based measurements (ABMs) extracted from one scientific publication

DBV	ABM	Type[1] and direction[2]	Reference
Voluntary culling (%)	Dirty calves	{+}	[3]
		(+)	[3]
Culling, overall (%)	Rising abnormal/ impaired	(+)	[3]
Culling for claw and leg disorders (%)	Lean calves	(−)	[3]
	Dirty calves	(−)	[3]
	Cows with injuries/ inflammation	{+}	[3]

[1] Type of associations indicated by ( ) for univariable associations and { } for multivariable associations.
[2] Direction of associations indicated by + for positive associations, − for negative associations

Table S5. Associations between data−based variables (DBVs) addressing veterinary diagnoses or treatments and animal−based measurements (ABMs) extracted from two scientific publications

DBV	ABM	Type[1] and direction[2]	Reference
Treatments per cow year (90d)	Cows with dirty udder	(+)	[4]
Treatments locomotor disorders (90d)	Avoidance distance, shyness	(+)	[4]
Treatments locomotor disorders (180d)	Lean or very lean cows	(−)	[4]
Proportion of locomotor disorders (365d)	Cows with dirty hindquarter	(+)	[4]
Proportion of locomotor disorders (90d)	Severely lame cows	(+)	[4]
Proportion of locomotor disorders (180d)	Rising abnormal/ impaired	(+)	[4]
Incidence of other feed−related diseases including gastrointestinal disturbances	Lean calves	{+}	[3]
	Lean young stock	(+)	[3]
	Dirty cows	{+}	[3]
Incidence of mastitis treatment	Lean or very lean cows	{+}	[3]

[1] Type of associations indicated by ( ) for univariable associations and { } for multivariable associations.
[2] Direction of associations indicated by + for positive associations, − for negative associations

Table S6. Associations between data−based variables (DBVs) based on slaughterhouse data and animal−based measurements (ABMs) extracted from three scientific publication

DBV	ABM	Type[1] and direction[2]	Reference
No remarks at slaughter (%)	Cows with injuries and inflammation	(−)	[3]
Proportion of abattoir remarks (180d)	impaired hair coat	(+)	[4]
Proportion of abattoir remarks (365d)	Lean or very lean cows	(−)	[4]
	impaired hair coat	(+)	[4]
Lean cows at slaughter (90d)	Integument alterations body	(−)	[4]
	Impaired hair coat	(+)	[4]
Lean cows at slaughter (365d)	Lean or very lean cows	(−)	[4]
	Cows with dirty hindquarter	(+)	[4]
	Impaired hair coat	(+)	[4]
Chronic inflammation (180d)	Integument alterations body	(−)	[4]
	Integument alterations tarsus	(+)	[4]
Chronic inflammation (365d)	Cows with dirty hindquarter	(−)	[4]
	Cows with dirty legs	(−)	[4]
	Cows with dirty udder	(−)	[4]
Liver abscesses (180d)	Cows with dirty legs	(−)	[4]
	Cows with dirty udder	(−)	[4]
Liver abscesses (365d)	Cows with dirty hindquarter	(−)	[4]
	Cows with dirty legs	(−)	[4]
Lung disorders (180d)	Integument alterations tarsus	(+)	[4]
Peritonitis (180d)	Avoidance distance, shyness	(+)	[4]

References

1. de Vries, M.; Bokkers, E.A.M.; van Schaik, G.; Engel, B.; Dijkstra, T.; de Boer, I.J.M. Exploring the value of routinely collected herd data for estimating dairy cattle welfare. *J. Dairy Sci.* **2014**, *97*, 715–730.
2. de Vries, M.; Bokkers, E.A.M.; van Schaik, G.; Engel, B.; Dijkstra, T.; de Boer, I.J.M. Improving the time efficiency of identifying dairy herds with poorer welfare in a population. *J. Dairy Sci.* **2016**, *99*, 8282–8296.
3. Sandgren, C.H.; Lindberg, A.; Keeling, L.J. Using a national dairy database to identify herds with poor welfare. *Anim. Welf.* **2009**, *18*, 523–532.
4. Otten, N.D.; Rousing, T.; Houe, H.; Thomsen, P.T.; Sørensen, J.T. Comparison of animal welfare indices in dairy herds based on different sources of data. *Anim. Welf.* **2016**, **25**, 207–215.
